# Inverse Association Between Resting-State Putamen Activity and Iowa Gambling Task Performance in Patients With Obsessive-Compulsive Disorder and Control Subjects

**DOI:** 10.3389/fpsyt.2022.836965

**Published:** 2022-05-13

**Authors:** Suguru Hasuzawa, Hirofumi Tomiyama, Keitaro Murayama, Aikana Ohno, Mingi Kang, Taro Mizobe, Kenta Kato, Akira Matsuo, Kazufumi Kikuchi, Osamu Togao, Tomohiro Nakao

**Affiliations:** ^1^Department of Neuropsychiatry, Graduate School of Medical Sciences, Kyushu University, Fukuoka, Japan; ^2^Graduate School of Human Environment Studies, Kyushu University, Fukuoka, Japan; ^3^Department of Clinical Radiology, Graduate School of Medical Sciences, Kyushu University, Fukuoka, Japan

**Keywords:** obsessive-compulsive disorder (OCD), Iowa Gambling Task (IGT), decision-making, putamen, resting-state functional MRI, fractional amplitude of low-frequency fluctuations (fALFF)

## Abstract

**Background:**

Symptoms of obsessive-compulsive disorder (OCD) have been conceptualized as manifestations of decision-making deficits. Patients with OCD exhibit impairment during the decision-making process, as assessed by the Iowa Gambling Task (IGT). This impairment is independent of clinical severity and disease progression. However, the association between the decision-making deficit and resting-state brain activity of patients with OCD has not been examined.

**Methods:**

Fifty unmedicated patients with OCD and 55 matched control subjects completed IGT. Resting-state brain activity was examined using the fractional amplitude of low-frequency fluctuations (fALFFs). fALFF analysis focused on the slow-4 and 5 bands. Group comparisons were performed to determine the association between IGT performance and fALFFs.

**Results:**

There was a significant group difference in the association between the IGT total net score and slow-4 fALFFs in the left putamen (voxel height threshold of *p* < 0.001; cluster size threshold of *p* < 0.05; family wise error-corrected). Higher putamen slow-4 fALFFs were correlated with lower IGT scores for OCD patients (*r* = −0.485; *p* < 0.0005) and higher IGT scores for control subjects (*r* = 0.402; *p* < 0.005). There was no group difference in the association between the IGT total net score and slow-5 fALFFs.

**Conclusions:**

These findings in unmedicated patients demonstrate the importance of resting-state putamen activity for decision-making deficit associated with OCD, as measured by IGT. The inverse correlation may be explained by the hypersensitive response of the putamen in patients with OCD.

## Introduction

Obsessive-compulsive disorder (OCD) is associated with chronic doubt and difficulty making decisions (1). Therefore, it has been suggested that OCD symptoms can be conceptualized as manifestations of a decision-making deficit. Neurocognitive research has highlighted decision-making deficits as the core feature of OCD (2–6). In this context, decision-making refers to the ability of an individual to process environmental information and choose an action that can yield a benefit. A benefit is achieved after the action as an immediate reward or after a certain period of time as a long-term reward. According to learning theory, the process during which individuals learn by trial and error to distinguish long-term rewards from immediate rewards and adjust their behaviors to maximize the ultimate benefit is called reinforcement learning (7). The Iowa Gambling Task (IGT) (8) is a well-established neuropsychological test that can assess the decision-making ability through reinforcement learning. IGT has been widely used in studies evaluating the decision-making deficits of patients with OCD (6, 9–14).

According to a meta-analysis of IGT performance in patients with OCD (15), patients perform significantly worse than control subjects (CTL). Moreover, IGT performance in patients with OCD is clinically important because it predicts the therapeutic response to serotonin reuptake inhibitors (6, 12). However, IGT performance is not necessarily correlated with the severity of overt clinical symptoms as measured by scales such as the Yale–Brown Obsessive Compulsive Scale (Y-BOCS) and Hamilton Anxiety Rating Scale (HAM-A) (6, 13, 16–18). Cavedini et al. (2) compared the poor IGT performance in depression and OCD patients and argued that the impairment observed in patients with depression is a state characteristic related to depressive mood, whereas in patients with OCD, the impairment constitutes a trait characteristic unrelated to the severity of overt symptoms. Furthermore, Zhang et al. (19) examined IGT performance in three OCD patient groups (i.e., non-medicated patients, medicated patients, and remitted patients) and found that all three groups performed worse than CTL; therefore, they concluded that the poor IGT performance in patients with OCD is trait-related. Because IGT performance in patients with OCD indicates a stable and long-lasting deficit that is not proportional to the severity of clinical phenotypes, some have regarded this performance as an endophenotype candidate for this disorder (9, 18–22).

Numerous studies have evaluated the brain activity during IGT performed by healthy individuals (23–28). IGT performance correlates with the activity of the frontal regions, including the ventromedial prefrontal cortex (vmPFC), dorsolateral prefrontal cortex (dlPFC), orbitofrontal cortex (OFC), supplementary motor area, and pre-supplementary motor area, as well as the striatum, insula, anterior cingulate cortex (ACC), and posterior cingulate cortex. These regions are involved in reward information processing. Regarding the neurobiological hypothesis of OCD, the cortico-striatal-thalamo-cortical (CSTC) loop model (29) is widely accepted, and the regions related to IGT performance largely overlap with the CSTC loops.

Nevertheless, neuroimaging studies evaluating the IGT performance in patients with OCD are limited. To the best of our knowledge, only two such studies have been conducted (30, 31), which examined the brain activity during each phase of the task; patients showed underactivation in the striatum and the frontal regions, including the vmPFC, dlPFC, and OFC, compared to controls during all three phases (i.e., outcome anticipation, decision-making, and outcome presentation). However, both of these studies have limitations. First, they targeted only adolescent patients with OCD and included patients using medication. Second, they used a shortened version of the IGT to reduce the stress that participants would experience during the simultaneous task performance and image acquisition. The administration of the shortened IGT version in the two studies resulted in no group differences in the IGT total net score.

If the poor IGT performance in patients with OCD is an endophenotype candidate for this disorder, then the abnormality might be detected in a purer form during the resting state, which is free from task load and symptom provocation. The aforementioned neuroimaging studies on IGT performance have focused mainly on task-evoked brain activation. However, if the poor IGT performance that occurs with OCD is derived from any trait-related problem, aberrations may be found in the intrinsic brain activity at rest. Moreover, performing neurocognitive assessments separately from image acquisition has the advantage of being less stressful for participants. In this case, it is not necessary to use a shortened version of IGT. Regarding other psychiatric disorders that can involve decision-making deficits (for example, pathological gambling, substance abuse, and eating disorders), some studies have revealed associations between impaired IGT performance and aberrant resting-state brain activity (32–34). However, no studies have examined the association between IGT performance and resting-state brain activity in patients with OCD.

Resting-state brain activity can be measured by the amplitude of low-frequency fluctuations (ALFFs) or fractional ALFFs (fALFFs) (35, 36). These measurements enable the assessment of the entire brain without a priori selection of regions of interest. The fALFFs comprise a relative measure of the ALFFs and are defined as the total power within a specific frequency band divided by the total power of the whole detectable frequency range. Compared to the measurement of ALFFs, which are susceptible to pulsatile artifacts, fALFF measurements have higher sensitivity and specificity (35, 36). Furthermore, in recent studies, low-frequency fluctuations were divided into several independent bands (slow-5, 0.01–0.027 Hz; slow-4, 0.027–0.073 Hz; slow-3, 0.073–0.198 Hz; slow-2, 0.198–0.25 Hz), and the features of various psychiatric disorders were investigated according to the properties of each band (37–41). Evidence has shown that the slow-2 and 3 bands are susceptible to physiological noise, such as cardiac signals, whereas the slow-4 and 5 bands can sensitively reveal neural activity (36). Slow-4 fluctuations are most robust in the basal ganglia and represent dopaminergic activities (36, 37, 42), whereas slow-5 fluctuations are more dominant than slow-4 fluctuations in the prefrontal cortices (especially in the ventromedial parts) (36, 37).

Abnormalities in fALFFs that occur with OCD have been investigated by numerous studies, but inconsistent results have been observed (40, 43–50). This inconsistency could be attributed to several factors, such as including patients using medications (40, 43, 47) and focusing exclusively on female patients (46). In contrast, Gao et al. (45) and Yang et al. (49) compared a large number of unmedicated patients with OCD and CTL and found significant group differences in fALFFs in the dlPFC, putamen, and superior frontal gyrus, as well as a negative correlation between functional connectivity in the striato-thalamic junction and symptom severity evaluated with the Y-BOCS. Because the CSTC loop model is supported by these findings of fALFF studies, it seems likely that resting-state CSTC abnormalities are related to decision-making deficit in OCD.

Here, we conducted a fALFF study of the resting-state brain activity in a large number of unmedicated adult patients with OCD and examined its relationship with IGT performance; then, we compared the results with those of CTL. Furthermore, we examined differences in fALFFs in whole-brain voxels of patients with OCD and CTL by considering the possibility of abnormalities related to decision-making deficit occurring in OCD without showing a direct correlation with IGT scores. As discussed previously, poor IGT performance in patients with OCD is thought to reflect a relatively invariable cognitive impairment, independent of the emotional disturbance (anxiety) severity. This suggests the involvement of the dorsal/cognitive part of the CSTC loops in poor IGT performance. Among the diverse brain regions involved in IGT performance in healthy individuals, the dlPFC and dorsal striatum belong to the dorsal CSTC loop. These are key regions for cognitive processing that allow individuals to learn to achieve large long-term rewards while incurring small immediate losses (51–56). In addition, the aforementioned fALFF studies on a large sample of patients with OCD (45, 49) have found abnormalities in these regions. Therefore, we hypothesized that the abnormalities responsible for the decision-making deficit associated with OCD would be found in these regions. Because slow-4 and 5 fALFFs sensitively reflect the neuroactivity of the basal ganglia and prefrontal cortices, respectively, we conducted a fALFF analysis, focusing mainly on these two bands.

## Materials and Methods

### Subjects

A total of 105 participants were recruited for this study, including 50 medication-free patients with OCD and 55 control subjects matched for age and sex. All patients with OCD were diagnosed using the Structured Clinical Interview for Diagnostic and Statistical Manual of Mental Disorders, fourth edition (DSM-IV), Axis I Disorders (patient edition), and fulfilled the DSM-IV criteria. We ensured that none of the participants met the criteria for any current comorbid Axis I disorder and that all participants also fulfilled the Diagnostic and Statistical Manual of Mental Disorders, fifth edition (DSM-5), criteria for OCD. The control subjects were recruited from the local community and interviewed according to the Structured Clinical Interview for DSM-IV (non-patient edition). None of the participants reported history of any psychiatric disorders. Candidates with a history of significant head injury, epilepsy, or intellectual disability were excluded.

This study was conducted in accordance with the principles of the Declaration of Helsinki. Ethical approval was obtained from the Kyushu University Ethics Committee. The study details were explained to all participants, and written informed consent was obtained from all participants.

### Clinical Assessment

The participants were clinically assessed in the same manner as performed during previous studies (57–59). The Japanese version of the Y-BOCS (60) was used to assess the global severity of OCD symptoms, while the Hamilton Rating Scale for Anxiety (HAM-A) (61) and the Hamilton Rating Scale for Depression (HAM-D, 17-item version) (62) were used to quantify the degrees of anxiety and depression. The Japanese version of the National Adult Reading Test (63) was used to measure the verbal intelligence quotient (IQ). Demographic and clinical data were statistically analyzed using the chi-square test, Student's *t*-test, and Mann-Whitney U test to detect group differences between the OCD and CTL groups.

### Neuropsychological Assessment

IGT was performed according to the original procedures developed by Bechara et al. (8); however, the hypothetical money was converted from United States dollars to Japanese yen. The participants selected cards from four decks labeled A, B, C, and D. At the start of the game, the participants were told that a loan of 200,000 yen was available. After picking a card, the participants would either win or lose some money. They could select cards from any deck, and the task was self-paced. The goal of the game was to win as much money as possible.

Card decks A and B were disadvantageous because they resulted in a net loss over time. Although they yielded larger immediate rewards, they inflicted larger penalties. In contrast, card decks C and D were advantageous because the total of the rewards was larger than that of the penalties. The participants were not informed of the risks of rewards and punishments in each deck or of the number of card selections allowed.

A total of 100 cards were selected to complete the task. Task performance was measured by the net score, which was calculated as the number of cards picked from the advantageous decks minus the number of cards picked from the disadvantageous decks.

### Image Data Acquisition and Preprocessing

Magnetic resonance imaging (MRI) data were acquired in the same manner as described previously (57–59). Image data were obtained using a 3.0-T MRI scanner (Achieva TX; Phillips Healthcare, Best, the Netherlands). T2^*^-weighted gradient-echo planar imaging sequence (echo time, 30 ms; repetition time, 2,500 ms; field of view, 212 × 212 mm; matrix, 64 × 64; slice thickness, 3.2 mm; flip angle, 80°) and high-resolution T1-weighted anatomical image (echo time, 3.8 ms; repetition time, 8.2 ms; field of view, 240 × 240 mm; flip angle, 8°; slice thickness, 1 mm; inversion time, 1,026 ms) results were acquired for each participant. A total of 240 real scans were obtained, which involved a 10-min real scan after an initial 10-s dummy scan. The participants were instructed to relax with their eyes open and to keep watching a presented gray cross during scanning. After MRI, the arousal level during the scan was evaluated using the Stanford-Sleepiness Scale.

Resting-state functional MRI data were analyzed using the CONN toolbox 20.b (64) with MATLAB R2020a. The first four functional volumes were removed, and the remaining 236 functional images were used for analysis. The images underwent slice-timing correction, realignment, and normalization in accordance with the standard Montreal Neurological Institute template. Six rigid body parameters were estimated for each subject. We applied the ART scrubbing procedure (https://www.nitrc.org/projects/artifact_detect/) to exclude image artifacts caused by head movement using the 97th percentile as the standard threshold of a normative sample (with thresholds for motion of 0.9 mm or global bold signal changes more than 5 standard deviations). There was no significant difference in the mean motion of the OCD and CTL groups (*t* = −1.226; *p* = 0.223). Functional images were smoothed using a Gaussian kernel with 6-mm full-width at half-maximum. Using the anatomical image of each participant, we created white matter and cerebrospinal fluid masks during the spatial processing steps. Signal noises from the white matter and cerebrospinal fluid were discerned by applying linear regression as a confounding effect (64). Then, fALFFs were computed using the preprocessed data. fALFFs were defined as the ratio of power in the low-frequency range to the total power in the entire frequency range for each individual voxel time series (35). The ratios of the power in the slow-5 (0.01–0.027 Hz) and slow-4 (0.027–0.073 Hz) ranges were calculated relative to the full frequency range (0–0.25 Hz), as previously described (36).

### Data Analysis

After calculating fALFFs of slow-4 and slow-5, we examined group differences in slow-4 and 5 fALFFs associated with the IGT total net score by using an analysis of covariance interaction model [statistical significance was set at a voxel height threshold of *p* < 0.001 and cluster-size threshold of *p* < 0.05; family-wise error (FWE) corrected (two-sided), with the Gaussian random field theory approach] while controlling for age, sex, and IQ, which have been reported to affect IGT performance (65–69). Additionally, we examined differences in fALFFs in whole brain voxels of the OCD and CTL groups while controlling for age, sex, and IQ [significance threshold set at a voxel height threshold of *p* < 0.001 and cluster size threshold of *p* < 0.05; FWE corrected (two-sided), with the Gaussian random field theory approach]. Furthermore, to strictly control the false-positive risk, we conducted a supplemental analysis of covariance by using a non-parametric permutation approach (5,000 times iterations), with a voxel height threshold of *p* < 0.001 and cluster-size threshold of *p* < 0.05 FWE corrected (70).

## Results

### Clinical Characteristics

The groups did not significantly differ in age, sex, handedness, or estimated verbal IQ (Table 1). All participants were medication-free for at least 4 weeks, and 16 patients with OCD were drug-naïve. The mean Y-BOCS score of the OCD group was 24.5 (standard deviation, ±5.51) (Table 1). During the acquisition of MRI data, no participant fell asleep, and there was no significant difference between the OCD and CTL groups in terms of the arousal level (Table 1).

**Table 1:** Demographic and clinical characteristics of the participants.

**Variable**	**OCD (*n* = 50)**	**CTL (*n* = 55)**	**Statistics**			
			***χ^2^***	***t***	**d*f***	***p*** **-value**
**Demographic and clinical characteristics**						
Sex, male/female	20/30	24/31	0.142		1	0.706
Hand, Edinburgh Handedness Inventory	68.00(61.09)	84.52(34.54)		1.67	75.773	0.100
Age, years	32.80(11.58)	34.2(11.66)		0.61	103	0.543
Estimated verbal IQ^a^	104.30(8.42)^b^	106.91(8.76)		1.54	103	0.127
HAM-D-17	3.98(4.04)	–				
HAM-A	4.98(6.35)	–				
Y-BOCS total	24.50(5.51)	–				
Y-BOCS obsessions	12.06(3.57)	–				
Y-BOCS compulsions	12.44(2.72)	–				
Onset, years	20.96(8.28)	–				
Illness duration, years	11.83(10.41)	–				
Stanford Sleepiness Scale	3.42(1.59)	3.31(1.43)		−0.26	102	0.793
IGT total net score	4.00(27.53)	11.09(23.43)		2.31	103	0.023^*^

*HAM-A, Hamilton Anxiety Scale; HAM-D, Hamilton Depression Scale; Y-BOCS, Yale-Brown Obsessive Compulsive Scale; IGT, Iowa Gambling Task*.

* *p < 0.05*.

a *Verbal IQ was estimated by the Japanese version of National Adult Reading Test (JART)*.

b *One participant did not complete JART*.

### IGT Performance

The groups showed a significant difference in IGT performance (Table 1). The IGT total net score was significantly lower for patients with OCD than for CTL. After controlling for age, sex, and verbal IQ, this result remained unchanged.

To evaluate the effects of symptom severity on task performance of the OCD group, we also analyzed the association between IGT total net scores and Y-BOCS scores (total, obsessions, and compulsions), HAM-D scores, and HAM-A scores. We found no significant association between IGT performance and clinical scores.

### fALFF Results

#### Associations Between IGT Performance and Slow-4 ALFFs

**Figure 1:**
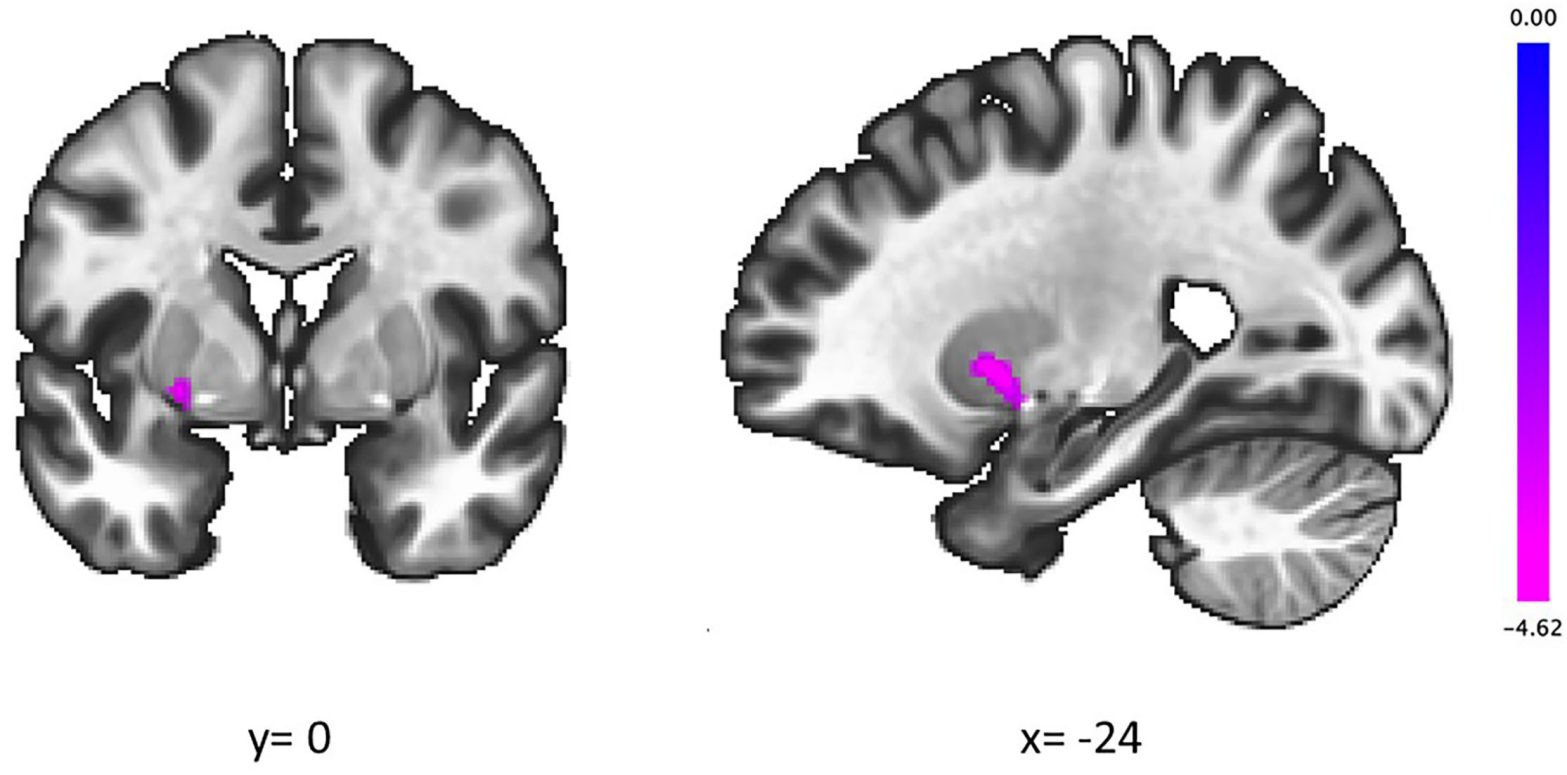
In the left putamen, higher slow-4 fALFFs were correlated with lower IGT scores in patients with OCD and higher IGT scores in CTL. Coordinates are given in MNI space. The color bar presents *t*-score. fALFFs, fractional amplitude of low-frequency fluctuations; IGT, Iowa Gambling Task; OCD, obsessive-compulsive disorder; CTL, control subjects.

**Figure 2:**
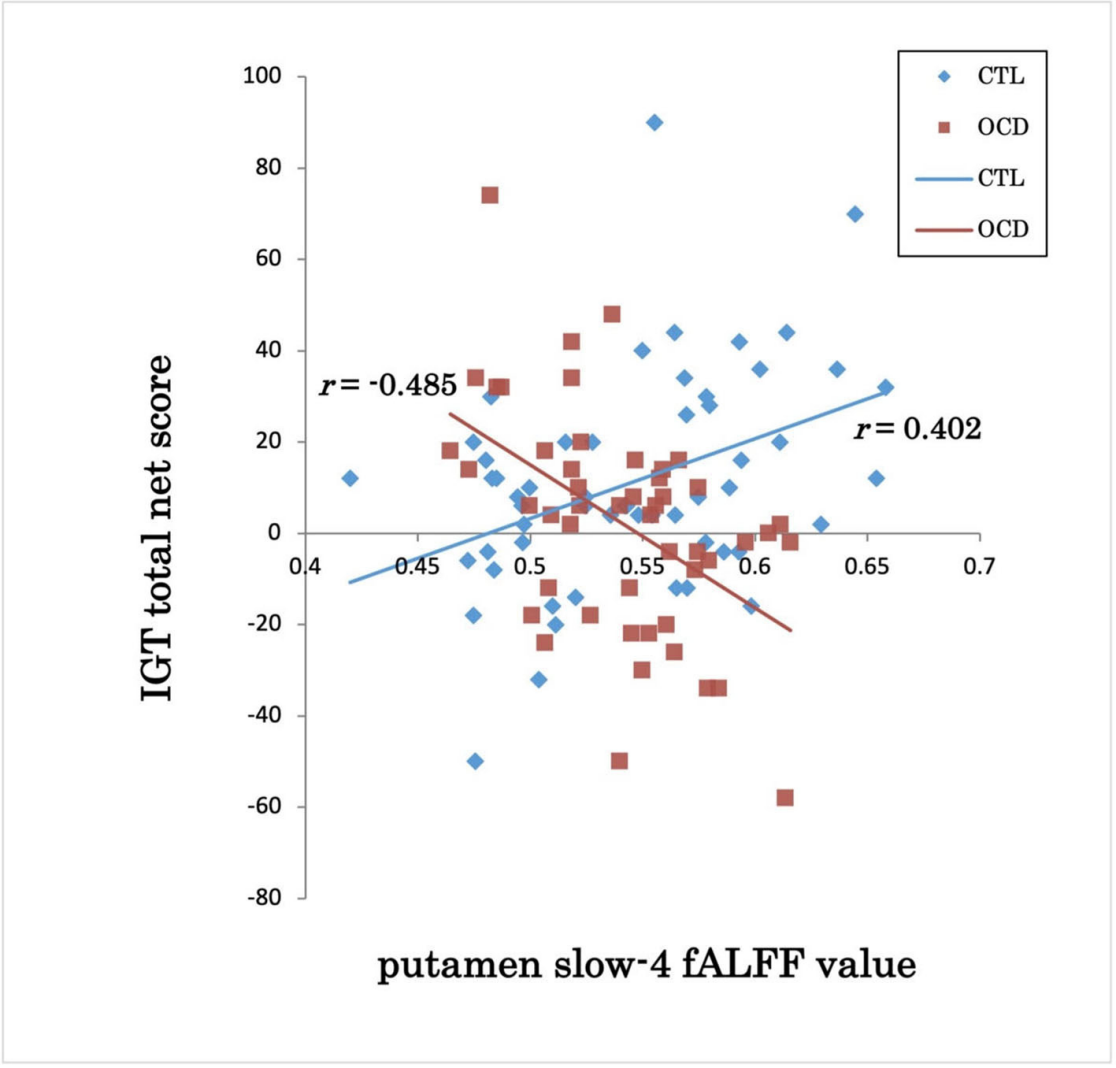
IGT total net score and putamen slow-4 fALFF value in OCD and CTL. IGT, Iowa Gambling Task; fALFFs, fractional amplitude of low-frequency fluctuations; OCD, obsessive-compulsive disorder; CTL, control subjects; *r*, Spearman's rank-order correlation coefficient.

The groups showed a significant difference in the association between the IGT total net score and fALFFs in the left putamen (voxel height threshold of *p* < 0.001 and cluster size threshold of *p* < 0.05; FWE corrected) (Figures 1, 2 and Table 2). In the OCD group, greater fALFF values in the left putamen were correlated with lower IGT total net scores (*r* = −0.485; *p* < 0.0005). The CTL group showed an inverse pattern, however, with greater putamen fALFFs being correlated with higher IGT total net scores (*r* = 0.402; *p* < 0.005). In the OCD group, there were no significant associations between the clinical assessment (Y-BOCS, HAM-D, HAM-A) scores and putamen fALFFs.

**Table 2:** Brain region where the association between IGT performance and slow-4 fALFFs was significantly different in OCD compared to CTL.

**Region**	**Ke**	***x***	***y***	***z***	***p*** **-FWE^a^**
L. putamen	72	−24	6	0	0.020839

a *Cluster-level corrected p < 0.05 FWE after applying a voxel height threshold of p < 0.001*.

*Peak coordinates are given in MNI space*.

*L, left; R, right; Ke, Cluster extent; fALFFs, fractional amplitude of low-frequency fluctuations; IGT, Iowa Gambling Task*.

In the supplemental analysis of covariance using a permutation-based cluster level correction, these results did not survive statistical significance.

#### Between-Group Differences in Slow-4 ALFFs

There were no significant differences between the slow-4 fALFFs in any brain region in the OCD and CTL groups.

#### Associations Between IGT Performance and Slow-5 ALFFs

There were no significant group differences in the association between slow-5 fALFFs and IGT performance.

#### Between-Group Differences in Slow-5 ALFFs

There were no significant differences between the slow-5 fALFFs in any brain region in the OCD and CTL groups.

## Discussion

We conducted a neuroimaging study using fALFFs to identify traits associated with poor IGT performance in patients with OCD. IGT performance was significantly worse in the OCD patient group than in the control group. There were no significant associations between IGT performance and the severity of psychiatric symptoms in patients with OCD.

Our main finding was the inverse correlation between putamen slow-4 fALFFs and IGT performance in patients with OCD and CTL. This intriguing phenomenon can be attributed to the aberrant putamen function during the reinforcement learning process. After examining the brain activity of patients with OCD during a probabilistic reversal learning task, Hauser et al. (71) reported that the patients showed significantly increased reward prediction error (RPE) responses in the ACC and putamen compared to CTL. RPEs, which have a crucial role in reinforcement learning, have been shown to be encoded by the mesolimbic dopamine system including the striatum and ACC (72–77). The findings of Hauser et al. (71) suggest that the hypersensitization of this RPE encoding is responsible for impaired decision-making in patients with OCD. Moreira et al. (78) examined the brain activity of patients with OCD during a gambling task and found results similar to those of Hauser et al. (71): when patients with OCD perceived unexpected losses, they demonstrated larger deactivation in the ACC and putamen than CTL. Because negative RPEs generally cause deactivation of mesolimbic dopamine neurons (79), the findings of Moreira et al. (78) suggest hypersensitization of the RPE response with OCD.

Our data showed that among patients with OCD, higher putamen fALFFs at rest were correlated with worse IGT performances. In general, high putamen fALFFs at rest are predictive of large phasic reactions during a learning task; studies of healthy subjects have demonstrated that for each brain region, there is a fixed correlation pattern for spontaneous activity at rest and task-evoked activation (80–84), and that resting-state striatal fALFFs reflect dopamine release levels in the area (85, 86). Therefore, with OCD, higher putamen fALFFs may result in larger hypersensitive responses to RPE. Because IGT performance depends on the sensitivity to RPEs (especially the sensitivity to negative RPEs) (28), OCD patients with higher putamen fALFFs will perform poorly. In contrast, among control subjects without such hypersensitivity, higher putamen activity will contribute to appropriate learning through RPEs and improve IGT performance.

Nevertheless, it is possible that the putamen function itself is intact in patients with OCD and that abnormalities exist elsewhere. Although a rostral part of the putamen was detected during our study, the posterior putamen, caudate, and nucleus accumbens are primarily involved in RPE processing (77, 87, 88). The OFC, vmPFC, and dlPFC have important roles in the valuation of action outcomes and guidance of goal-directed behavior (28, 89, 90). The putamen receives the results of information processing in these regions and automates learned action sequences as a habit (91–94). Habit refers to the automatic and outcome-insensitive repetition of certain action sequences (95–97). Previous studies on decision-making deficits with OCD have inferred that aberrant information processing in the OFC and dlPFC, implicated in shaping goal-directed behavior, leads patients to make choices that do not produce valuable outcomes (98–102). Although our data showed no significant difference between the CTL and OCD groups in terms of resting-state fALFF values, the possibility of aberrations in the OFC or dlPFC cannot be ruled out. If information processing in these regions is altered, then disadvantageous behaviors will be learned and fixed as habits through putamen activity. Therefore, in patients with OCD, higher putamen activity may facilitate fixation of disadvantageous behaviors, which results in poorer IGT performance. On the contrary, among control subjects, information processing in the OFC and dlPFC is intact and leads to the appropriate selection of beneficial behaviors. Consequently, the higher putamen activity of CTL facilitates the fixation of beneficial behaviors, which results in better IGT performance. However, the present results could not confirm this. Future research using seed-based analysis to investigate the association between functional connectivity from the PFC to the putamen and decision-making ability should be conducted to verify this second explanation.

To our knowledge, the correlation between putamen fALFFs and IGT performance in healthy individuals has not been reported previously. Nevertheless, Kambeitz et al. (103) reported that healthy individuals with higher striatal dopamine levels at rest had higher net IGT scores. Because putamen fALFF values are thought to reflect the actual dopamine level in the region (85, 86, 104), our results are consistent with those reported by Kambeitz et al. (103). Additionally, according to Kayser et al. (56) and Smith et al. (53), healthy individuals with lower putamen activity and those with lower dopamine synthesis capacity in the putamen showed higher choice impulsivity and preferred immediate rewards to long-term rewards. Therefore, our CTL group findings are supported by these studies.

In the present study, we hypothesized that the abnormalities responsible for poor IGT performance with OCD would be found in the dorsal CSTC loop with projections from the dlPFC to the thalamus via the dorsal striatum. However, contrary to our hypothesis, there were no significant differences in fALFFs in any brain region of the OCD and CTL groups. Previous studies that recruited a large number of unmedicated patients with OCD (45, 49) showed higher fALFFs in the dlPFC and putamen in the patient group than in the control group. This inconsistency might be attributable to differences in the various profiles of the examined OCD patient groups. Functional neuroimaging studies of patients with OCD showed that aberrant activation of the dlPFC and putamen observed in patient groups was associated with the duration of illness (105, 106). Increased putamen activation was also related to greater comorbidity with mood and anxiety disorders (105). Furthermore, Mataix-Cols et al. (107) suggested that abnormal activation of the dlPFC and putamen with OCD is specifically associated with the aggressive/checking symptom dimension. By considering and comparing these factors in the present study and in the studies by Yang et al. (49) and Gao et al. (45), it is apparent that the illness duration was longer in our patient group (11.83 ± 10.41 years) than in the patient group studied by Yang et al. (6.4 ± 5.2 years; Gao et al. did not report this information). Regarding comorbidity with mood and anxiety disorders, our patients had even lower HAM-D and HAM-A scores (3.98 ± 4.04 and 4.98 ± 6.35, respectively) than the patients studied by Gao et al. (45) (6.7 ± 4.2 and 7.8 ± 5.4, respectively) and Yang et al. (49) (7.56 ± 3.76 and 7.6 ± 3.5, respectively), thus indicating that our patients more accurately represent the core OCD group without comorbidities. Although we cannot compare symptom dimensions because of the lack of information, it is possible that the studies by Gao et al. (45) and Yang et al. (49) included more patients with aggressive/checking symptoms than our study included. All these differences in the profiles of patients with OCD might be responsible for the inconsistent results.

The aforementioned factors are also associated with the limitations of this study. First, the different symptom dimensions of OCD may have distinct neural correlates (106–108). For a more precise understanding of decision-making deficits with OCD, a large number of patients with each symptom dimension should be enrolled in a study to compare IGT performance between the groups.

Second, the present study was not free from medication effects. Although we recruited 50 patients with OCD who were medication-free for at least 4 weeks, only 16 of them were drug-naïve. For the remaining patients, the possible residual effects of previous medications could not be ruled out. Therefore, more drug-naïve patients should be recruited in future analyses.

Third, we recruited only patients who were diagnosed with OCD without any other Axis I disorder. However, OCD is highly comorbid with anxiety and depressive disorders (109). It is unclear whether our findings are valid for patients with such comorbidities. Future work should include OCD patients with comorbidities and compare their results with the results of a core OCD group and a group of CTL to evaluate decision-making deficits.

Fourth, it is also unclear to what extent our findings are specific to OCD. Poor IGT performance has been reported in several psychiatric disorders (32–34). To confirm that our findings are strictly specific to OCD, we should recruit patients with these psychiatric disorders and examine group differences in fALFFs associated with the IGT scores.

Fifth, as control subjects, we recruited individuals who had no past and current DSM disorder. However, according to Kessler et al. (108), about half of the general population will meet the criteria for a DSM disorder at some point in life. Given such a high potential risk, it would be desirable to perform detailed clinical assessments of control subjects and compare the results with those of the patient group.

Lastly, it is possible that our results may be affected by false-positive risk (70). When we applied the most conservative statistical threshold to control for multiple comparisons (using a non-parametric permutation approach, with a voxel height threshold of *p* < 0.001 and cluster-size threshold of *p* < 0.05 FWE corrected) (70, 109), our results could not survive statistical significance. We consider that the presently applied threshold (using the typical SPM based approach with Gaussian random field theory, with cluster defined threshold with a voxel height threshold of *p* < 0.001 and cluster-level threshold of *p* < 0.05 FWE corrected) is strict enough to control FWE, even when we take into account the problem raised by Eklund et al. (70). Many recent neuroimaging studies (110, 111) also used a similar threshold for multiple comparison correction. Nevertheless, the possibility of false-positive risk cannot be completely ruled out.

## Conclusions

We confirmed that unmedicated patients with OCD exhibited worse IGT performance than CTL. This poor performance was not correlated with the severity of clinical manifestations as assessed by the Y-BOCS or HAM-D. Furthermore, we examined group differences in fALFFs associated with the IGT scores of OCD patients and CTL. We found an inverse correlation between putamen slow-4 fALFFs and IGT performance in patients with OCD and CTL. Higher putamen fALFFs were correlated with lower IGT scores in patients with OCD and higher IGT scores in CTL. These findings highlight the importance of resting-state putamen activity for the processes of learning and decision-making as assessed by IGT. The inverse association detected in the present study is consistent with previously reported hypersensitive responses of the putamen to RPEs. Such abnormal functioning of the putamen may comprise a biological feature underlying aberrations in the CSTC loops that were observed during previous studies using task-related MRI to evaluate IGT performance in patients with OCD. If the perseveration tendency in OCD as assessed by IGT is related to the putamen function, the putamen as well as the network between the putamen and the prefrontal cortices may be targets for neuromodulatory treatments in OCD.

## Data Availability Statement

The original contributions presented in the study are included in the article/supplementary material, further inquiries can be directed to the corresponding author/s.

## Ethics Statement

The studies involving human participants were reviewed and approved by Kyushu University Ethics Committee. The patients/participants provided their written informed consent to participate in this study.

## Author Contributions

SH designed the study, collected data, and wrote the initial draft of the manuscript. HT and KM collected data, designed the study, and critically reviewed the manuscript. AO and MK contributed to analysis and interpretation of data. TM, KKa, AM, KKi, and OT contributed to data collection. TN critically reviewed the manuscript. All authors approved the final version of the manuscript.

## Funding

This work was supported by the Japan Society for the Promotion of Science (JSPS) KAKENHI Grant Numbers (C) JP21K07547 and JP19K08076.

## Conflict of Interest

The authors declare that the research was conducted in the absence of any commercial or financial relationships that could be construed as a potential conflict of interest.

## Publisher's Note

All claims expressed in this article are solely those of the authors and do not necessarily represent those of their affiliated organizations, or those of the publisher, the editors and the reviewers. Any product that may be evaluated in this article, or claim that may be made by its manufacturer, is not guaranteed or endorsed by the publisher.

## References

[1] Foa EB Mathews A Abramowitz JS Amir N Przeworski A Riggs DS. Do patients with obsessive-compulsive disorder have deficits in decision-making? Cognit Ther Res. (2003) 27:431–45. 10.1023/A:1025424530644

[2] Cavedini P Gorini A Bellodi L. Understanding obsessive-compulsive disorder: focus on decision making. Neuropsychol Rev. (2006) 16:3–15. 10.1007/s11065-006-9001-y 16708289

[3] Dittrich WH Johansen T. Cognitive deficits of executive functions and decision-making in obsessive-compulsive disorder. Scand J Psychol. (2013) 54:393–400. 10.1111/sjop.12066 23841985

[4] Nestadt G Kamath V Maher BS Krasnow J Nestadt P Wang Y. Doubt and the decision-making process in obsessive-compulsive disorder. Med Hypotheses. (2016) 96:1–4. 10.1016/j.mehy.2016.09.010 27959266 PMC6013040

[5] Sachdev PS Malhi GS. Obsessive-compulsive behaviour: a disorder of decision-making. Aust N Z J Psychiatry. (2005) 39:757–63. 10.1080/j.1440-1614.2005.01680.x 16168033

[6] Cavedini P Riboldi G D'annucci A Belotti P Cisima M Bellodi L. Decision-making heterogeneity in obsessive-compulsive disorder: ventromedial prefrontal cortex function predicts different treatment outcomes. Neuropsychologia. (2002) 40:205–211. 10.1016/S0028-3932(01)00077-X 11640942

[7] Sutton RS Barto AG. Reinforcement learning an introduction second edition. A Bradford Book. (1998). 10.1109/TNN.1998.712192

[8] Bechara A Damasio AR Damasio H Anderson SW. Insensitivity to future consequences following damage to human prefrontal cortex. Cognition. (1994) 50:7–15. 10.1016/0010-0277(94)90018-3 8039375

[9] Cavedini P Zorzi C Piccinni M Cavallini MC Bellodi L. Executive dysfunctions in obsessive-compulsive patients and unaffected relatives: searching for a new intermediate phenotype. Biol Psychiatry. (2010) 67:1178–84. 10.1016/j.biopsych.2010.02.012 20381015

[10] Grassi G Pallanti S Righi L Figee M Mantione M Denys D. Think twice: Impulsivity and decision making in obsessive-compulsive disorder. J Behav Addict. (2015) 4:263–72. 10.1556/2006.4.2015.039 26690621 PMC4712760

[11] Grassi G Makris N Pallanti S. Addicted to compulsion: assessing three core dimensions of addiction across obsessive-compulsive disorder and gambling disorder. CNS Spectr. (2020) 25:392–401. 10.1017/S1092852919000993 31106718 PMC6864250

[12] Cavedini P Bassi T Zorzi C Bellodi L. The advantages of choosing antiobsessive therapy according to decision-making functioning. J Clin Psychopharmacol. (2004) 24:628–31. 10.1097/01.jcp.0000144889.51072.03 15538125

[13] da Rocha FF Alvarenga NB Malloy-Diniz L Corrêa H. Decision-making impairment in obsessive-compulsive disorder as measured by the Iowa Gambling Task. Arq Neuropsiquiatr. (2011) 69:642–7. 10.1590/S0004-282X2011000500013 21877034

[14] Martoni RM Brombin C Nonis A Salgari GC Buongiorno A Cavallini MC. Evaluating effect of symptoms heterogeneity on decision-making ability in obsessive-compulsive disorder. Psychiatry Clin Neurosci. (2015) 69:402–10. 10.1111/pcn.12264 25522816

[15] Nisticò V de Angelis A Erro R Demartini B Ricciardi L. Obsessive-compulsive disorder and decision making under ambiguity: a systematic review with meta-analysis. Brain Sci. (2021) 11:1–23. 10.3390/brainsci11020143 33499211 PMC7912249

[16] Krishna R Udupa S George CM Kumar KJ Viswanath B Kandavel T. Neuropsychological performance in OCD: a study in medication-naïve patients. Prog Neuro Psychopharmacol Biol Psychiatry. (2011) 35:1969–76. 10.1016/j.pnpbp.2011.09.009 21967733

[17] Lawrence NS Wooderson S Mataix-Cols D David R Speckens A Phillips ML. Decision making and set shifting impairments are associated with distinct symptom dimensions in obsessive-compulsive disorder. Neuropsychology. (2006) 20:409–19. 10.1037/0894-4105.20.4.409 16846259

[18] Zhang L Dong Y Ji Y Zhu C Yu F Ma H. Dissociation of decision making under ambiguity and decision making under risk: a neurocognitive endophenotype candidate for obsessive-compulsive disorder. Prog Neuro Psychopharmacol Biol Psychiatry. (2015) 57:60–8. 10.1016/j.pnpbp.2014.09.005 25315855

[19] Zhang L Dong Y Ji Y Tao R Chen X Ye J Zhang L Yu F Zhu C Wang K. Trait-related decision making impairment in obsessive-compulsive disorder: Evidence from decision making under ambiguity but not decision making under risk. Sci Rep. (2015) 5:17312. 10.1038/srep17312 26601899 PMC4658550

[20] Benzina N Mallet L Burguière E N'Diaye K Pelissolo A. Cognitive dysfunction in obsessive-compulsive disorder. Curr Psychiatry Rep. (2016) 18:80. 10.1007/s11920-016-0720-3 27423459

[21] Chamberlain SR Blackwell AD Fineberg NA Robbins TW Sahakian BJ. The neuropsychology of obsessive compulsive disorder: the importance of failures in cognitive and behavioural inhibition as candidate endophenotypic markers. Neurosci Biobehav Rev. (2005) 29:399–419. 10.1016/j.neubiorev.2004.11.006 15820546

[22] Bora E. Meta-analysis of neurocognitive deficits in unaffected relatives of obsessive-compulsive disorder (OCD): Comparison with healthy controls and patients with OCD. Psychol Med. (2020) 50:1257–66. 10.1017/S0033291720001634 32476632

[23] Fukui H Murai T Fukuyama H Hayashi T Hanakawa T. Functional activity related to risk anticipation during performance of the Iowa gambling task. Neuroimage. (2005) 24:253–9. 10.1016/j.neuroimage.2004.08.028 15588617

[24] Northoff G Grimm S Boeker H Schmidt C Bermpohl F Heinzel A. Affective judgment and beneficial decision making: Ventromedial prefrontal activity correlates with performance in the Iowa Gambling Task. Hum Brain Mapp. (2006) 27:572–87. 10.1002/hbm.20202 16372256 PMC6871437

[25] Lin CH Chiu YC Cheng CM Hsieh JC. Brain maps of Iowa gambling task. BMC Neurosci. (2008) 9:72. 10.1186/1471-2202-9-72 18655719 PMC2518922

[26] Li X Lu ZL D'Argembeau A Ng M Bechara A. The Iowa Gambling Task in fMRI images. Hum Brain Mapp. (2010) 31:410–23. 10.1002/hbm.20875 19777556 PMC2826566

[27] Lawrence NS Jollant F O'Daly O Zelaya F Phillips ML. Distinct roles of prefrontal cortical subregions in the iowa gambling task. Cerebral Cortex. (2009) 19:1134–43. 10.1093/cercor/bhn154 18787233

[28] Christakou A Brammer M Giampietro V Rubia K. Right ventromedial and dorsolateral prefrontal cortices mediate adaptive decisions under ambiguity by integrating choice utility and outcome evaluation. Journal of Neuroscience. (2009) 29:11020–8. 10.1523/JNEUROSCI.1279-09.2009 19726660 PMC6665528

[29] Saxena S Brody AL Schwartz JM Baxter LR. Neuroimaging and frontal-subcortical circuitry in obsessive-compulsive disorder. Br J Psychiatry. (1998) 173:26–37. 10.1192/S0007125000297870 9829024

[30] Norman LJ Carlisi CO Christakou A Murphy CM Chantiluke K Giampietro V. Frontostriatal dysfunction during decision making in attention-deficit/hyperactivity disorder and obsessive-compulsive disorder. Biol Psychiatry Cogn. Neurosci. Neuroimag. (2018) 3:694–703. 10.1016/j.bpsc.2018.03.009 29706587 PMC6278892

[31] Carlisi CO Norman L Murphy CM Christakou A Chantiluke K Giampietro V. Shared and disorder-specific neurocomputational mechanisms of decision-making in autism spectrum disorder and obsessive-compulsive disorder. Cerebral Cortex. (2017) 27:5804–16. 10.1093/cercor/bhx265 29045575 PMC6919268

[32] Ho MC Chen VCH Chao SH Fang CT Liu YC Weng JC. Neural correlates of executive functions in patients with obesity. PeerJ. (2018) 6:e5002. 10.7717/peerj.5002 29910989 PMC6003388

[33] Qiu YW Han LJ Lv XF Jiang GH Tian JZ Zhuo FZ. Regional homogeneity changes in heroin-dependent individuals: Resting-state functional MR imaging study. Radiology. (2011) 261:551–9. 10.1148/radiol.11102466 21875854

[34] Pettorruso M Martinotti G Cocciolillo F de Risio L Cinquino A di Nicola M. Striatal presynaptic dopaminergic dysfunction in gambling disorder: a 123I-FP-CIT SPECT study. Addict Biol. (2019) 24:1077–86. 10.1111/adb.12677 30226290

[35] Zou QH Zhu CZ Yang Y Zuo XN Long XY Cao QJ. An improved approach to detection of amplitude of low-frequency fluctuation (ALFF) for resting-state fMRI: Fractional ALFF. J Neurosci Methods. (2008) 172:137–41. 10.1016/j.jneumeth.2008.04.012 18501969 PMC3902859

[36] Zuo XN di Martino A Kelly C Shehzad ZE Gee DG Klein DF. The oscillating brain: Complex and reliable. Neuroimage. (2010) 49:1432–45. 10.1016/j.neuroimage.2009.09.037 19782143 PMC2856476

[37] Yu R Chien YL Wang HLS Liu CM Liu CC Hwang TJ. Frequency-specific alternations in the amplitude of low-frequency fluctuations in schizophrenia. Hum Brain Mapp. (2014) 35:627–37. 10.1002/hbm.22203 23125131 PMC6869729

[38] Egorova N Veldsman M Cumming T Brodtmann A. Fractional amplitude of low-frequency fluctuations (fALFF) in post-stroke depression. NeuroImage Clin. (2017) 16:116–24. 10.1016/j.nicl.2017.07.014 28794972 PMC5537409

[39] Belleau EL Kremens R Ang YS Pisoni A Bondy E Durham K. Reward functioning abnormalities in adolescents at high familial risk for depressive disorders. Biol Psychiatry Cogn Neurosci Neuroimag. (2021) 6:270–9. 10.1016/j.bpsc.2020.08.016 33160881 PMC7936004

[40] Giménez M Guinea-Izquierdo A Villalta-Gil V Martínez-Zalacaín I Segalàs C Subirà M. Brain alterations in low-frequency fluctuations across multiple bands in obsessive compulsive disorder. Brain Imaging Behav. (2017) 11:1690–706. 10.1007/s11682-016-9601-y 27771857

[41] Han Y Wang J Zhao Z Min B Lu J Li K. Frequency-dependent changes in the amplitude of low-frequency fluctuations in amnestic mild cognitive impairment: a resting-state fMRI study. Neuroimage. (2011) 55:287–95. 10.1016/j.neuroimage.2010.11.059 21118724

[42] Xue SW Li D Weng XC Northoff G Li DW. Different neural manifestations of two slow frequency bands in resting functional magnetic resonance imaging: a systemic survey at regional, interregional, and network levels. Brain Connect. (2014) 4:242–55. 10.1089/brain.2013.0182 24456196

[43] Cheng Y Xu J Nie B Luo C Yang T Li H Lu J Xu L Shan B Xu X. Abnormal resting-state activities and functional connectivities of the anterior and the posterior cortexes in medication-naïve patients with obsessive-compulsive disorder. PLoS ONE. (2013) 8:e67478. 10.1371/journal.pone.0067478 23840714 PMC3696097

[44] Qiu L Fu X Wang S Tang Q Chen X Cheng L. Abnormal regional spontaneous neuronal activity associated with symptom severity in treatment-naive patients with obsessive-compulsive disorder revealed by resting-state functional MRI. Neurosci Lett. (2017) 640:99–104. 10.1016/j.neulet.2017.01.024 28104431

[45] Gao J Zhou Y Yang X Luo J Meng F Zheng D Li Z. Abnormalities within and beyond the cortico-striato-thalamo-cortical circuitry in medication-free patients with OCD revealed by the fractional amplitude of low-frequency fluctuations and resting-state functional connectivity. Neurosci Lett. (2019) 712:134449. 10.1016/j.neulet.2019.134449 31470044

[46] Meng Z Zhang Z Fan Q Li Y. Altered fractional amplitude of low frequency fluctuations in unmedicated female patients with obsessive-compulsive disorder. In: Annual International Conference of the IEEE Engineering in Medicine and Biology - Proceedings. (2018). p. 1144–7. 10.1109/EMBC.2018.8512490 30440592

[47] Li K Zhang H Yang Y Zhu J Wang B Shi Y Li X Meng Z Lv L Zhang H. Abnormal functional network of the thalamic subregions in adult patients with obsessive-compulsive disorder. Behav. Brain Res. (2019) 371:111982. 10.1016/j.bbr.2019.111982 31141727

[48] Zhang H Wang B Li K Wang X Li X Zhu J. Altered functional connectivity between the cerebellum and the cortico-striato-thalamo-cortical circuit in obsessive-compulsive disorder. Front Psychiatry. (2019) 10:522. 10.3389/fpsyt.2019.00522 31396115 PMC6667674

[49] Yang X Hu X Tang W Li B Yang Y Gong Q. Intrinsic brain abnormalities in drug-naive patients with obsessive-compulsive disorder: a resting-state functional MRI study. J Affect Disord. (2019) 245:861–8. 10.1016/j.jad.2018.11.080 30699871

[50] Yang X Hu X Tang W Li B Yang Y Gong Q Huang X. Multivariate classification of drug-naive obsessive-compulsive disorder patients and healthy controls by applying an SVM to resting-state functional MRI data. BMC Psychiatry. (2019) 19:210. 10.1186/s12888-019-2184-6 31277632 PMC6612132

[51] Tanaka CS Doya K Okada G Ueda K Okamoto Y Yamawaki S. Prediction of immediate and future rewards differentially recruits cortico-basal ganglia loops. Nat Neurosci. (2004) 7:887–93. 10.1038/nn1279 15235607

[52] Tanaka SC Schweighofer N Asahi S Shishida K Okamoto Y Yamawaki S Doya K. Serotonin differentially regulates short- and long-term prediction of rewards in the ventral and dorsal striatum. PLoS ONE. (2007) 2:e1333. 10.1371/journal.pone.0001333 18091999 PMC2129114

[53] Smith CT Wallace DL Dang LC Aarts E Jagust WJ D'Esposito M. Modulation of impulsivity and reward sensitivity in intertemporal choice by striatal and midbrain dopamine synthesis in healthy adults. J Neurophysiol. (2016) 115:1146–56. 10.1152/jn.00261.2015 26683066 PMC4808128

[54] van den Bos W Rodriguez CA Schweitzer JB McClure SM. Connectivity strength of dissociable striatal tracts predict individual differences in temporal discounting. J Neurosci. (2014) 34:10298–310. 10.1523/JNEUROSCI.4105-13.2014 25080591 PMC4577570

[55] van den Bos W Rodriguez CA Schweitzer JB McClure SM. Adolescent impatience decreases with increased frontostriatal connectivity. Proc Natl Acad Sci USA. (2015) 112:E3765–74. 10.1073/pnas.1423095112 26100897 PMC4517266

[56] Kayser AS Allen DC Navarro-Cebrian A Mitchell JM Fields HL. Dopamine, corticostriatal connectivity, and intertemporal choice. J. Neurosci. (2012) 32:9402–9. 10.1523/JNEUROSCI.1180-12.2012 22764248 PMC6622221

[57] Tomiyama H Nakao T Murayama K Nemoto K Ikari K Yamada S. Dysfunction between dorsal caudate and salience network associated with impaired cognitive flexibility in obsessive-compulsive disorder: a resting-state fMRI study. NeuroImage: Clin. (2019) 24:102004. 10.1016/j.nicl.2019.102004 31622840 PMC6812295

[58] Murayama K Tomiyama H Tsuruta S Ohono A Kang M Hasuzawa S. Aberrant resting-state cerebellar-cerebral functional connectivity in unmedicated patients with obsessive-compulsive disorder. Front Psychiatry. (2021) 12:659616. 10.3389/fpsyt.2021.659616 33967861 PMC8102723

[59] Tomiyama H Murayama K Nemoto K Tomita M Hasuzawa S Mizobe T. Increased functional connectivity between presupplementary motor area and inferior frontal gyrus associated with the ability of motor response inhibition in obsessive–compulsive disorder. Hum. Brain Mapp. (2021) 43:974–84. 10.1002/hbm.25699 34816523 PMC8764470

[60] Nakajima T Nakamura M Taga C Toshihiko Kinoshita S Okajima Y Hanada M. Reliability and validity of the Japanese version of the Yale-Brown Obsessive-Compulsive Scale. Psychiatry Clin Neurosci. (1995) 49:121–6. 10.1111/j.1440-1819.1995.tb01875.x 8726128

[61] Hamilton M. The assessment of anxiety states by rating. Br J Med Psychol. (1959) 32:50–5. 10.1111/j.2044-8341.1959.tb00467.x 13638508

[62] Hamilton M. A rating scale for depression. J Neurol Neurosurg Psychiatry. (1960) 23:56–62. 10.1136/jnnp.23.1.56 14399272 PMC495331

[63] Matsuoka K Uno M Kasai K Koyama K Kim Y. Estimation of premorbid IQ in individuals with Alzheimer's disease using Japanese ideographic script (Kanji) compound words: Japanese version of national adult reading test. Psychiatry Clin Neurosci. (2006) 60:332–9. 10.1111/j.1440-1819.2006.01510.x 16732750

[64] Whitfield-Gabrieli S Nieto-Castanon A. Conn: a functional connectivity toolbox for correlated and anticorrelated brain networks. Brain Connect. (2012) 2:125–41. 10.1089/brain.2012.0073 22642651

[65] Cauffman E Shulman EP Steinberg L Claus E Banich MT Graham S. Age differences in affective decision making as indexed by performance on the iowa gambling task. Dev Psychol. (2010) 46:193–207. 10.1037/a0016128 20053017

[66] Mata R Josef AK Samanez-Larkin GR Hertwig R. Age differences in risky choice: a meta-analysis. Ann N Y Acad Sci. (2011) 1235:18–29. 10.1111/j.1749-6632.2011.06200.x 22023565 PMC3332530

[67] van den Bos R Homberg J de Visser L, A. critical review of sex differences in decision-making tasks: focus on the iowa gambling task. Behav Brain Res. (2013) 238:95–108. 10.1016/j.bbr.2012.10.002 23078950

[68] Demaree HA Burns KJ DeDonno MA. Intelligence, but not emotional intelligence, predicts Iowa Gambling Task performance. Intelligence. (2010) 38:249–54. 10.1016/j.intell.2009.12.004

[69] Webb CA DelDonno S Killgore WDS. The role of cognitive versus emotional intelligence in Iowa gambling task performance: what's emotion got to do with it? Intelligence. (2014) 44:112–9. 10.1016/j.intell.2014.03.008 25635149 PMC4307614

[70] Eklund A Nichols TE Knutsson H. Cluster failure: Why fMRI inferences for spatial extent have inflated false-positive rates. Proc Natl Acad Sci USA. (2016) 113:7900–5. 10.1073/pnas.1602413113 27357684 PMC4948312

[71] Hauser TU Iannaccone R Dolan RJ Ball J Hättenschwiler J Drechsler R. Increased fronto-striatal reward prediction errors moderate decision making in obsessive-compulsive disorder. Psychol Med. (2017) 47:1246–58. 10.1017/S0033291716003305 28065182

[72] Schultz W Apicella P Scarnati E Ljungbergc T. Neuronal activity in monkey ventral striatum related to the expectation of reward. J Neurosci. (1992) 72:4595–610. 10.1523/JNEUROSCI.12-12-04595.1992 1464759 PMC6575755

[73] Breiter HC Aharon I Kahneman D Dale A Shizgal P. Functional imaging of neural responses to expectancy and experience of monetary gains and losses. Neuron. (2001) 30:619–639. 10.1016/S0896-6273(01)00303-8 11395019

[74] Holroyd CB Coles MGH. The neural basis of human error processing: reinforcement learning, dopamine, and the error-related negativity. Psychol Rev. (2002) 109:679–709. 10.1037/0033-295X.109.4.679 12374324

[75] McClure SM Berns GS Read Montague P. Temporal prediction errors in a passive learning task activate human striatum. Neuron. (2003) 38:339–46. 10.1016/S0896-6273(03)00154-5 12718866

[76] O'Doherty JP Dayan P Friston K Critchley H Dolan RJ. Temporal difference models and reward-related learning in the human brain. Neuron. (2003) 28:329–37. 10.1016/S0896-6273(03)00169-7 12718865

[77] Pessiglione M Seymour B Flandin G Dolan RJ Frith CD. Dopamine-dependent prediction errors underpin reward-seeking behaviour in humans. Nature. (2006) 442:1042–5. 10.1038/nature05051 16929307 PMC2636869

[78] Moreira PS Macoveanu J Marques P Coelho A Magalhães R Siebner HR. Altered response to risky decisions and reward in patients with obsessive–compulsive disorder. J Psychiatry Neurosci. (2020) 45:98–107. 10.1503/jpn.180226 31509362 PMC7828903

[79] Schultz W Dayan P Read Montague P. A Neural Substrate of Prediction and Reward. Neuropsychopharmacology. (1997) 28:56. 10.1126/science.275.5306.1593 9054347

[80] Zou Q Ross TJ Gu H Geng X Zuo XN Hong LE. Intrinsic resting-state activity predicts working memory brain activation and behavioral performance. Hum Brain Mapp. (2013) 34:3204–15. 10.1002/hbm.22136 22711376 PMC6870161

[81] Mennes M Zuo XN Kelly C di Martino A Zang YF Biswal B. Linking inter-individual differences in neural activation and behavior to intrinsic brain dynamics. Neuroimage. (2011) 54:2950–9. 10.1016/j.neuroimage.2010.10.046 20974260 PMC3091620

[82] Fox MD Snyder AZ Zacks JM Raichle ME. Coherent spontaneous activity accounts for trial-to-trial variability in human evoked brain responses. Nat Neurosci. (2006) 9:23–5. 10.1038/nn1616 16341210

[83] Liu X Zhu XH Chen W. Baseline BOLD correlation predicts individuals' stimulus-evoked BOLD responses. Neuroimage. (2011) 54:2278–86. 10.1016/j.neuroimage.2010.10.001 20934521 PMC3006639

[84] Fox MD Snyder AZ Vincent JL Raichle ME. Intrinsic fluctuations within cortical systems account for intertrial variability in human behavior. Neuron. (2007) 56:171–84. 10.1016/j.neuron.2007.08.023 17920023

[85] Meyer B Mann C Götz M Gerlicher A Saase V Yuen KSL. Increased neural activity in mesostriatal regions after prefrontal transcranial direct current stimulation and L-DOPA administration. J Neurosci. (2019) 39:5326–35. 10.1523/JNEUROSCI.3128-18.2019 31043485 PMC6607760

[86] Nakamura Y Nakamura Y Pelosi A Djemai B Debacker C Hervé D Girault JA Tsurugizawa T. fMRI detects bilateral brain network activation following unilateral chemogenetic activation of direct striatal projection neurons. NeuroImage (2020) 220:117079. 10.1016/j.neuroimage.2020.117079 32585345

[87] Haruno M Kawato M. Different neural correlates of reward expectation and reward expectation error in the putamen and caudate nucleus during stimulus-action-reward association learning. J Neurophysiol. (2006) 95:948–59. 10.1152/jn.00382.2005 16192338

[88] Haruno M Kuroda T Doya K Toyama K Kimura M Samejima K. Neural correlate of reward-based behavioral learning in caudate nucleus: a functional magnetic resonance imaging study of a stochastic decision task. J Neurosci. (2004) 24:1660–5. 10.1523/JNEUROSCI.3417-03.2004 14973239 PMC6730455

[89] Riceberg JS Shapiro ML. Orbitofrontal cortex signals expected outcomes with predictive codes when stable contingencies promote the integration of reward history. J Neurosci. (2017) 37:2010–21. 10.1523/JNEUROSCI.2951-16.2016 28115481 PMC5338752

[90] Valentin VV Dickinson A O'Doherty JP. Determining the neural substrates of goal-directed learning in the human brain. J Neurosci. (2007) 27:4019–26. 10.1523/JNEUROSCI.0564-07.2007 17428979 PMC6672546

[91] Gillan CM Robbins TW. Goal-directed learning and obsessive–compulsive disorder. Philos Transac Royal Soc B Biol Sci. (2014) 369: 0130475. 10.1098/rstb.2013.0475 25267818 PMC4186229

[92] Tricomi E Balleine BW O'Doherty JP. A specific role for posterior dorsolateral striatum in human habit learning. Eur J Neurosci. (2009) 29:2225–32. 10.1111/j.1460-9568.2009.06796.x 19490086 PMC2758609

[93] Tremel JJ Laurent PA Wolk DA Wheeler ME Fiez JA. Neural signatures of experience-based improvements in deterministic decision-making. Behav Brain Res. (2016) 315:51–65. 10.1016/j.bbr.2016.08.023 27523644 PMC5017924

[94] Everitt BJ Robbins TW. Neural systems of reinforcement for drug addiction: from actions to habits to compulsion. Nat Neurosci. (2005) 8:1481–9. 10.1038/nn1579 16251991

[95] Daw ND Niv Y Dayan P. Uncertainty-based competition between prefrontal and dorsolateral striatal systems for behavioral control. Nat Neurosci. (2005) 8:1704–11. 10.1038/nn1560 16286932

[96] de Wit S Dickinson A. Associative theories of goal-directed behaviour: a case for animal-human translational models. Psychol Res. (2009) 73:463–76. 10.1007/s00426-009-0230-6 19350272 PMC2694930

[97] Yin HH Ostlund SB Balleine BW. Reward-guided learning beyond dopamine in the nucleus accumbens: The integrative functions of cortico-basal ganglia networks. Eur J Neurosci. (2008) 28:1437–48. 10.1111/j.1460-9568.2008.06422.x 18793321 PMC2756656

[98] Vaghi MM Vértes PE Kitzbichler MG Apergis-Schoute AM van der Flier FE Fineberg NA. Specific frontostriatal circuits for impaired cognitive flexibility and goal-directed planning in obsessive-compulsive disorder: evidence from resting-state functional connectivity. Biol Psychiatry. (2017) 81:708–17. 10.1016/j.biopsych.2016.08.009 27769568 PMC6020061

[99] Vaghi MM Moutoussis M Váša F Kievit RA Hauser TU Vértes PE. Compulsivity is linked to reduced adolescent development of goal-directed control and frontostriatal functional connectivity. Proc Natl Acad Sci USA. (2020) 117:25911–22. 10.1073/pnas.1922273117 32989168 PMC7568330

[100] Vaghi MM Hampshire A Fineberg NA Kaser M Brühl AB Sahakian BJ. Hypoactivation and dysconnectivity of a frontostriatal circuit during goal-directed planning as an endophenotype for obsessive-compulsive disorder. Biol Psychiatry Cogn Neurosci Neuroimag. (2017) 2:655–63. 10.1016/j.bpsc.2017.05.005 29167834 PMC5684958

[101] van den Heuvel OA Veltman DJ Groenewegen HJ Cath DC van Balkom AJ van Hartskamp J. Frontal-striatal dysfunction during planning in obsessive-compulsive disorder. Arch Gen Psychiatry. (2005) 62:301–10. 10.1001/archpsyc.62.3.301 15753243

[102] Wood J Ahmari SE. A framework for understanding the emerging role of corticolimbic-ventral striatal networks in OCD-associated repetitive behaviors. Front Syst Neurosci. (2015) 9:171. 10.3389/fnsys.2015.00171 26733823 PMC4681810

[103] Kambeitz J. la Fougère C, Werner N, Pogarell O, Riedel M, Falkai P, Ettinger U. Nicotine-dopamine-transporter interactions during reward-based decision making. Eur Neuropsychopharmacol. (2016) 26:938–47. 10.1016/j.euroneuro.2016.03.011 27112968

[104] Hu ML Zong XF Zheng JJ Pantazatos SP Miller JM Li ZC. Short-term effects of risperidone monotherapy on spontaneous brain activity in first-episode treatment-naïve schizophrenia patients: a longitudinal fMRI study. Sci Rep. (2016) 6:34287. 10.1038/srep34287 27698361 PMC5048418

[105] Thorsen AL Hagland P Radua J Mataix-Cols D Kvale G Hansen B. Emotional processing in obsessive-compulsive disorder: a systematic review and meta-analysis of 25 functional neuroimaging studies. Biol Psychiatry Cogn Neurosci Neuroimag. (2018) 3:563–71. 10.1016/j.bpsc.2018.01.009 29550459 PMC5994188

[106] del Casale A Kotzalidis GD Rapinesi C Serata D Ambrosi E Simonetti A. Functional neuroimaging in obsessive-compulsive disorder. Neuropsychobiology. (2011) 64:61–85. 10.1159/000325223 21701225

[107] Mataix-Cols D Wooderson S Lawrence N Brammer MJ Speckens A Phillips ML. Distinct neural correlates of washing, checking, and hoarding symptom dimensions in obsessive-compulsive disorder. Arch Gen Psychiatry. (2004) 61:564–76. 10.1001/archpsyc.61.6.564 15184236

[108] Kessler RC Berglund P Demler O Ma R Jin MA Merikangas KR. Lifetime prevalence and age-of-onset distributions of DSM-IV disorders in the national comorbidity survey replication. Arch Gen Psychiatry. (2005) 62:593–602. 10.1001/archpsyc.62.6.593 15939837

[109] Cox RW Chen G Glen DR Reynolds RC Taylor PA FMRI. Clustering in AFNI: false-positive rates redux. Brain Connect. (2017) 7:152–71. 10.1089/brain.2016.0475 28398812 PMC5399747

[110] Seidel M Geisler D Borchardt V King JA Bernardoni F Jaite C. Evaluation of spontaneous regional brain activity in weight-recovered anorexia nervosa. Transl. Psychiatry. (2020) 10:395. 10.1038/s41398-020-01081-0 33177499 PMC7658198

[111] Tomiyama H Murayama K Nemoto K Hasuzawa S Mizobe T Kato K. Alterations of default mode and cingulo-opercular salience network and frontostriatal circuit: a candidate endophenotype of obsessive-compulsive disorder. Prog Neuro Psychopharmacol Biol Psychiatry. (2022) 116:110516. 10.1016/j.pnpbp.2022.110516 35108587

